# Izalontamab (SI-B001), a Novel EGFRxHER3 Bispecific Antibody in Patients with Locally Advanced or Metastatic Epithelial Tumor: Results from First-in-Human Phase I/Ib Study

**DOI:** 10.1158/1078-0432.CCR-25-0206

**Published:** 2025-04-21

**Authors:** Jinhui Xue, Yuxiang Ma, Yuanyuan Zhao, Yongsheng Wang, Wei Hong, Yan Huang, Yunpeng Yang, Wenfeng Fang, Shaodong Hong, Yang Zhang, Qianwen Liu, Yi Zhu, Hai Zhu, Sa Xiao, Li Zhang, Hongyun Zhao

**Affiliations:** 1 State Key Laboratory of Oncology in South China, Guangdong Key Laboratory of Nasopharyngeal Carcinoma Diagnosis and Therapy, Guangdong Provincial Clinical Research Center for Cancer, Sun Yat-sen University Cancer Center, Guangzhou, China.; 2 West China Hospital of Sichuan University, Chengdu, China.; 3 Zhejiang Cancer Hospital, Zhejiang, China.; 4 Sichuan Baili Pharmaceutical Co. Ltd., Chengdu, China.

## Abstract

**Purpose:**

Izalontamab (SI-B001) is a novel EGFR×HER3 bispecific antibody. This first-in-human phase I study presents the safety and pharmacokinetics of izalontamab.

**Patients and Methods:**

Previously treated patients with locally advanced or metastatic epithelial tumors were enrolled in the dose-escalation or dose-expansion phases. The dose-escalation phase consisted of an accelerated titration and a “3 + 3” design with nine dose levels from 0.4 to 28.0 mg/kg. The dose-expansion phase included five dose levels from 6.0 to 21.0 mg/kg. Izalontamab was administered intravenously weekly or every 2 weeks in a 4-week cycle. Available pretreatment specimens were obtained to explore the relationship between EGFR/HER3 expression and efficacy.

**Results:**

Sixty patients were enrolled. Among the 60 enrolled patients, 49 had non–small cell lung cancer (NSCLC), 6 had nasopharyngeal cancer, 3 had head and neck cancer squamous cell carcinoma, and 2 had other types of cancer. The most common treatment-related adverse events were rash (42%), paronychia (25%), and infusion-related reactions (23%). No drug-related death occurred. Izalontamab displayed a nonlinear pharmacokinetic behavior, and clearance at steady state seemed to be approaching a dose-independent value at 6 mg/kg and above. The best response included two confirmed partial responses in patients with NSCLC and head and neck cancer squamous cell carcinoma; 18 patients had stable disease, including NSCLC (*n* = 17) and colorectal cancer (*n* = 1). The recommended phase II dose for izalontamab was determined as 9 to 16 mg/kg weekly.

**Conclusions:**

Izalontamab was well tolerated and demonstrated preliminary antitumor activity in patients with locally advanced or metastatic epithelial tumors, supporting it as a promising therapeutic candidate for combination therapies, with a phase III study currently underway.

**Translational Relevance**

This study shows that izalontamab (SI-B001), a novel EGFR×HER3 bispecific antibody, has a manageable safety profile and preliminary antitumor activity in patients with locally advanced or metastatic epithelial tumors. Izalontamab shows potential for combination with existing therapies, with a phase III study currently ongoing.

## Introduction

The EGFR and receptor tyrosine protein kinase ERBB3 (HER3) are receptor tyrosine kinases belonging to the ErbB family, known for their role in activating signaling pathways and contributing to tumorigenesis and tumor progression. HER3 is a special receptor with limited kinase activity and requires heterodimerization with other family members for signaling, whereas anti-HER3 treatments have failed to attract significant attention in the past. Despite this, HER3 is expressed across a variety of solid tumors, and its overexpression is associated with poor survival (1–3). Additionally, HER3 amplification is associated with acquired resistance to current treatments (4, 5). With this perspective, targeting both EGFR and HER3 simultaneously in combination therapies could potentially enhance antitumor efficacy (6).

Bispecific antibodies, designed to simultaneously bind two antigens or epitopes, are constantly being developed and optimized to improve drug efficacy and mitigate toxicity and have advanced enormously over the past two decades (7). Despite this, the development of bispecific antibodies targeting the ErbB family has been relatively slow. To date, only bispecific antibodies targeting EGFR/c-Met (amivantamab) and HER2/HER3 (zenocutuzumab) have been approved (8, 9), with no bispecific antibodies targeting EGFR/HER3 having been approved yet. Bispecific antibodies targeting EGFR/HER3 are expected to become a new treatment option for solid tumors.

Izalontamab (SI-B001) is a novel EGFR×HER3 bispecific antibody that targets EGFR and HER3. It consists of an anti-EGFR human IgG1 antibody fused with two anti-HER3 human single-chain fragment variables via the glycine–serine linker (10). SI-B001 binds to EGFR×EGFR homodimers and blocks their downstream pathways (11). Preclinical studies showed that izalontamab has encouraging antitumor efficacy as well as acceptable tolerance in several xenograft models, including colon cancer, head and neck squamous cell carcinoma (HNSCC), and esophageal cancer (11).

We conducted this multicenter phase I trial to assess the safety, MTD, and preliminary efficacy of izalontamab in patients with advanced or metastatic epithelial tumors who failed standard treatment. We also explored potential biomarkers for efficacy by immunofluorescence staining.

## Patients and Methods

This was a phase I, multicenter, open-label study with two stages: a dose-escalation phase and a schedule expansion phase. This study was approved by the Ethics Committee of Sun Yat-sen University Cancer Center and West China Hospital, Sichuan University. This trial was conducted according to the tenets of the Declaration of Helsinki and Good Clinical Practices. The Clinicaltrials.gov registration number is NCT04603287.

### Patients

This multicenter phase I trial included patients with advanced solid tumors who failed standard treatment. The inclusion criteria were (i) at least 18 years of age; (ii) pathologically confirmed advanced unresectable or metastatic solid tumors, progression after standard treatment, no standard treatment option available, or could not tolerate the standard treatment; (iii) measurable lesions at baseline by imaging per the RECIST (version 1.1); and (iv) an Eastern Cooperative Oncology Group performance status score of 0 or 1. The exclusion criteria were (i) history of severe heart disease, (ii) history of grade 3 lung disease defined according to Common Terminology Criteria for Adverse Events version 5.0 or a history of interstitial lung disease; and (iii) symptoms of active central nervous system metastasis, except for patients with stable brain metastasis. Detailed inclusion and exclusion criteria are provided in Supplementary Methods S1. Written informed consent was obtained from all patients.

### Study design

The dose-escalation phase included an accelerated titration at 0.4 and 1.2 mg/kg, followed by a traditional “3 + 3” design at 3.0, 6.0, 9.0, 12.0, 16.0, 21.0, and 28.0 mg/kg. The dose-expansion phase evaluated five dose levels: 6.0, 9.0, 12.0, 16.0, and 21.0 mg/kg. Izalontamab was administered intravenously weekly or every 2 weeks (for 28 mg/kg only) in a 4-week cycle. As a single-arm trial, this study involved no randomization or blinding. Treatment was continued until disease progression, unacceptable toxicity, lost to follow-up, patient dropout, or other reasons for discontinuation (e.g., withdrawal of consent or death)

The primary objectives of phase Ia were to evaluate the safety and tolerability of izalontamab to determine the MTD and dose-limiting toxicity (DLT). The primary objectives of phase Ib are to further assess the safety and tolerability and determine the recommended phase II dose (RP2D). The secondary objectives include safety evaluation, preliminary efficacy, pharmacokinetics (PK), and immunogenicity of izalontamab. MTD is defined as the dose at which the DLT occurred in no more than 33% of the patients within 28 days after the first dosing. DLTs included grade 3 or severe adverse events (AE) related to izalontamab, except for grade 3 skin toxicity that has resolved to grade ≤2 after treatment within 7 days; grade 3 nausea, vomiting, diarrhea, or anorexia that has resolved to grade ≤2 after treatment within 7 days; grade 3 hypomagnesemia; grade 3 fatigue that has resolved to grade ≤1 within 7 days; hair loss at any level; and infusion-related reactions. The RP2D was selected based on its safety, antitumor activity, and PK analysis. The exploratory objective was to evaluate the relationship between EGFR/HER3 protein expression levels and efficacy.

### Assessments

Safety assessments, including laboratory metrics, were conducted on a planned schedule. Treatment-emergent AEs (TEAE) were graded according to Common Terminology Criteria for Adverse Events, and treatment-related AEs (TRAE) were judged by investigators. AEs were monitored throughout the study, extending until 28 days after the last dose or initiation of other antitumor treatment.

CT or MRI examination was performed every two cycles ±3 days until the initiation of new antineoplastic therapy, disease progression, withdrawal of informed consent, dropout, lost to follow-up, or death, whichever occurred first. Tumor response was evaluated according to RECIST version 1.1. The antitumor activity assessment included objective response rate [ORR defined as the proportion of patients achieving complete response or partial response (PR)], disease control rate (DCR, comprising patients with PRs or stable disease), and progression-free survival (PFS, defined as the time from treatment initiation to disease progression or death).

### PK

Blood samples for PK analysis were collected before infusion (within 4 hours), after infusion (15 minutes after infusion), and 2, 4, 6, 12, 24, 48, 96, and 168 hours after the first and third doses at cycle 1. Only preinfusion (within 4 hours) and postinfusion (15 minutes after infusion) blood samples were collected for the rest of the treatment schedules. PK parameters including half-life (t_1/2_), time to reach maximum plasma concentration (T_max_), maximum concentration (C_max_), AUC–concentration curve from 0 to the last detectable concentration collection time t (AUC_0–t_) and infinite time (AUC_0–∞_), volume of distribution (Vd), and clearance (CL) were calculated using a noncompartmental model with the “Linear Up Log Down” method using Phoenix WinNonlin version 8.4.0 (Certara, L.P.), based on the actual blood sampling time points. The immunogenicity of therapeutic proteins was detected using the antidrug antibody (ADA).

### Biomarker analyses

Analyses of HER3 and EGFR expression were performed in available pretreatment tumor tissues by IHC assay in Teddy Clinical Research Laboratory Limited, Shanghai. The paraffin sections were dewaxed and were autoclaved with Tris-EDTA repair solution. Primary antibodies anti-HER3 (Abcam, Cat. #ab93739, RRID: AB_10563976, 1:50 dilution) and anti-EGFR (Ventana Medical Systems Cat. #790-4347, RRID: AB_2617183, 1:1,600 dilution) were added on slides, and a drop of PBS was added as a blank control for background assessment. After washing, the biotinylated detection reagent was added dropwise, followed by the addition of diaminobenzidine (DAB) colorant. The samples were restained with hematoxylin, dehydrated, clarified, and sealed with neutral resin. Then the protein expression was detected using the OptiView DAB IHC Detection Kit. The score result was obtained by multiplying the cell staining intensity with the percentage of positive cells. H-score was calculated using the formula H-score = 1 × (1+%) + 2 × (2+%) +3 × (3+%). The staining intensity was graded as follows: 0 (absence of staining), 1+ (weak staining), 2+ (moderate-intensity staining), and 3+ (strong staining); (1+%), (2+%), and (3+%) are the values of percentages of positive cells in three intensity levels.

### Statistical analysis

As a phase I study evaluating safety and MTD, a formal power calculation was not applicable. The sample size was estimated based on the accelerated titration plus the traditional “3 + 3” design. The safety analysis set included patients who received at least one dose of izalontamab. The efficacy analysis set included patients who received at least one dose of izalontamab and had baseline and postmedication efficacy evaluation data, except those who dropped out unrelated to the study. The PK analysis set included subjects who have used at least one study drug and have at least one calculable PK parameter. Safety was summarized descriptively. Point estimates and two-sided 95% exact binomial confidence intervals (95% CI) were used to describe ORR and DCR. PFS was summarized descriptively using the Kaplan–Meier method, and the 95% CIs for all median survival times were calculated. All statistical analyses were performed using Statistical Analysis System (RRID: SCR_008567, version 9.4), R version 4.1.2, and Phoenix WinNonlin (RRID: SCR_024504, version 8.4.0).

## Results

### Baseline patient demographics and disease characteristics

**Figure 1.**
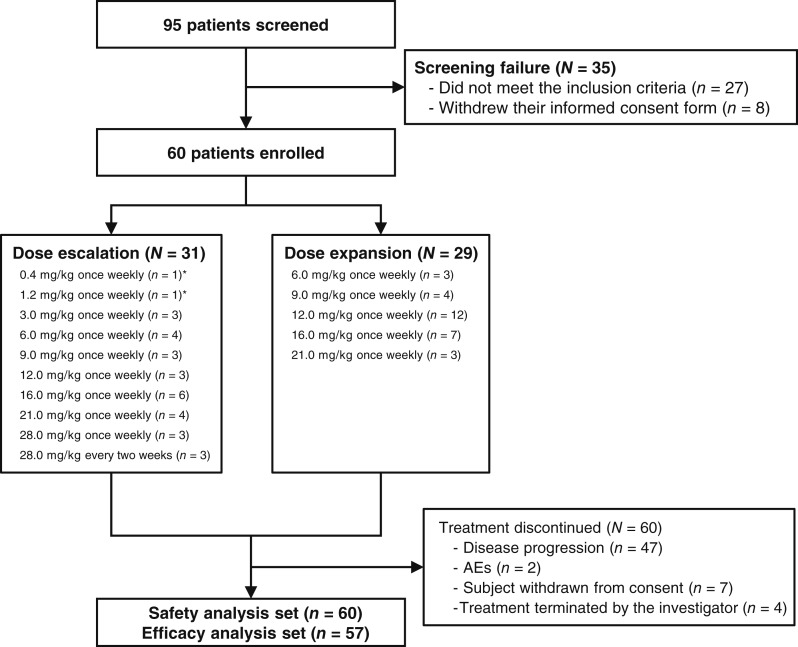
Study design of the clinical study. *Doses of 0.4 and 1.2 mg/kg were administered using an accelerated titration design.

From April 2020 to December 2022, a total of 95 patients were screened for eligibility, and 60 (31 in phase Ia and 29 in phase Ib) patients were eventually enrolled in izalontamab monotherapy (Fig. 1). Among the 60 enrolled patients, 49 had non–small cell lung cancer (NSCLC), 6 had nasopharyngeal cancer, 3 had HNSCC, and 2 had other types of cancer.

Among the 49 enrolled patients with lung cancer, 28 had squamous cell carcinoma and 21 had adenocarcinoma. Of the adenocarcinoma cases, 18 patients were identified with EGFR mutations. Patient characteristics are listed in Table 1 and Supplementary Table S1 (Supplementary Table S2 for Study Representativeness). Overall, the median age was 57 years, and most participants were male (75%). Most patients (36/60, 60%) have progressed after receiving three or more lines of prior therapy. Twenty (33%) patients had previously received anti-EGFR treatment, including EGFR tyrosine kinase inhibitors, cetuximab and nimotuzumab. The data cutoff for analysis was August 30, 2024, and the median follow-up was 27.5 (20.4 and 36.5) months.

**Table 1.** Baseline demographic and clinical characteristics.

Characteristic	Ia (*n* = 31)	Ib (*n*= 29)	Total (*N* = 60)
Age (years), median (range)	55.0 (23.0–70.0)	58.0 (32.0–74.0)	57.0 (23.0–74.0)
Male	23 (74)	22 (76)	45 (75)
Smoking history			
Never	16 (52)	11 (38)	27 (45)
Current	0	2 (7)	2 (3)
Former	15 (48)	16 (55)	31 (52)
ECOG			
0	11 (36)	6 (21)	17 (28)
1	20 (65)	23 (79)	43 (72)
Tumor type			
Lung cancer	24 (77)	25 (86)	49 (82)
Nasopharyngeal cancer	4 (13)	2 (7)	6 (10)
HNSCC	2 (7)	1 (4)	3 (5)
Other types	1 (3)	1 (4)	2 (3)
Stage			
III	0	3 (10)	3 (5)
IV	31 (100)	26 (90)	57 (95)
Prior line of therapy			
1 line	4 (13)	5 (17)	9 (15)
2 line	6 (19)	9 (31)	15 (25)
3 line and above	21 (68)	15 (52)	**36 (60)**

Data are *n* (%).

Abbreviation: ECOG, Eastern Cooperative Oncology Group.

### Safety

In total 60 patients were included in the safety analysis. The median drug exposure time (IQR) was 50.5 (23.5–112.5) days. In the dose-escalation cohort, 31 cases were included in the DLT analysis set. No DLT was observed, and the MTD was not reached.

TRAEs were observed in 92% (*n* = 55) of all participants. The most common TRAEs were rash (42%), paronychia (25%), and infusion-related reactions (23%; Table 2). Nine (15%) grade ≥3 TRAEs were observed, including infusion-related reaction (*n* = 2), decreased lymphocyte counts (*n* = 2), hypercalcemia (*n* = 2), rash (*n* = 1), hypomagnesemia (*n* = 1), and neutropenia (*n* = 1). Three (5%) patients discontinued treatment, and one (2%) had dose reduction due to TRAEs. No drug-related death occurred. AEs of different dose groups are shown in Supplementary Table S3. TEAEs occurring in more than 10% are summarized in Supplementary Table S4. The most common TEAEs included rash [27 (45%)], proteinuria [21 (35%)], and paronychia [17 (28%)].

**Table 2.** Most common TRAEs.

TRAE	All (*N* = 60)			
	Grade 1	Grade 2	Grade ≥3	Any grade
Rash	19 (32)	5 (8)	1 (2)	25 (42)
Paronychia	8 (13)	7 (12)	0	15 (25)
IRR	6 (10)	6 (10)	2 (3)	14 (23)
Pruritus	9 (15)	2 (3)	0	11 (18)
Pyrexia	10 (17)	1 (2)	0	11 (18)
Lymphocyte count decreased	4 (7)	3 (5)	2 (3)	9 (15)
Stomatitis	9 (15)	0	0	9 (15)
Milia	5 (8)	2 (3)	0	7 (12)
Asthenia	5 (8)	0	0	5 (8)
Hypomagnesemia	3 (5)	1 (2)	1 (2)	5 (8)
Alanine aminotransferase increased	4 (7)	0	0	4 (7)
Decreased appetite	2 (3)	2 (3)	0	4 (7)
Dermatitis acneiform	3 (5)	1 (2)	0	4 (7)
Diarrhea	4 (7)	0	0	4 (7)
Hematuria	4 (7)	0	0	4 (7)
Hypokalemia	3 (5)	1 (2)	0	4 (7)
Hypophosphatemia	4 (7)	0	0	4 (7)
Leukopenia	3 (5)	1 (2)	0	4 (7)
Nausea	2 (3)	2 (3)	0	4 (7)
Proteinuria	3 (5)	1 (2)	0	4 (7)
Aspartate aminotransferase increased	3 (5)	0	0	3 (5)
Hypercalcemia	1 (2)	0	2 (3)	3 (5)
Neutropenia	1 (2)	1 (2)	1 (2)	3 (5)
Vomiting	1 (2)	2 (3)	0	3 (5)

TRAEs in ≥5% of patients are shown in the table.

Abbreviation: IRR, infusion-related reaction.

Skin rash was mild and mostly limited to grade 1 (32% of patients) or grade 2 (8% of patients). No QT prolongation was observed with izalontamab from ECG analyses.

### PK

**Figure 2.**
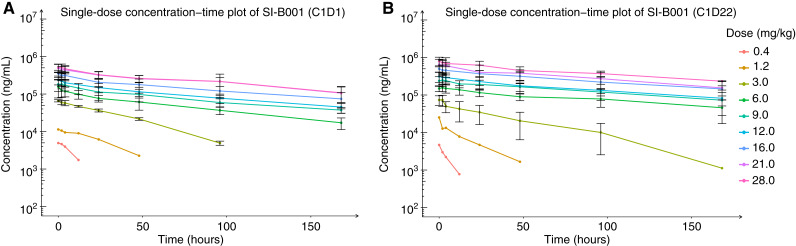
Dose concentration–time plot of izalontamab concentration at different dosage regimens. **A,** Izalontamab concentration–time profile at C1D1. **B,** Izalontamab concentration–time profile at C1D22. C1D1, cycle 1 day 1; C1D22, cycle 1 day 22. Mean and SD are shown.

Fifty-five patients were included in the PK analysis. Izalontamab displayed a nonlinear PK behavior in serum following infusions, with concentration–time profiles demonstrating a faster clearance at low dose levels (0.4–3 mg/kg; Fig. 2). The clearance at the steady state of izalontamab decreased with the increasing dose and seemed to be approaching a dose-independent value of 0.02 to 0.03 L/hours at 6 mg/kg and above, indicating target saturation. The volume of distribution at steady state (Vss) was low (range, 2.9–3.9 L) at 6 mg/kg and above. Following izalontamab administration at 6.0, 9.0, 12.0, 16.0, and 21.0 mg/kg, the mean C_max_ (coefficient of variation%, CV%) values were 159.7 (21.8%), 264.1 (28.3%), 322.4 (31.7%), 502.5 (20%), and 679.5 (44.9%) mg/L; and the mean C_trough_ (CV%) values were 31.1 (25.8%), 61.5 (22.4%), 78.8 (64.1%), 130.1 (26.5%), and 138.4 (54.5%) mg/L. The accumulation index increased with increasing dose and approached a steady state (range from 1.3–1.5) at 6 mg/kg and above. The concentration–time curves and steady-state PK parameters are shown in Supplementary Fig. S1 and Supplementary Table S5.

A total of 60 patients were available for immunogenicity tests. Three of 60 (5%) patients were drug-induced ADA positive during drug administration (Supplementary Table S6). The ADA had no effect on drug exposure.

### RP2D

As no DLT was observed, the MTD was not determined. RP2D determination was based on the safety and PK analyses. According to the PK data, the half-life of izalontamab is about 4 to 5 days at 6 mg/kg and above, which supports weekly dosing. The clearance of izalontamab above 6 mg/kg reached a dose-independent steady state, indicating that a dosage greater than 6 mg/kg is required to achieve receptor saturation (Supplementary Fig. S1). The incidence of grade ≥3 TRAEs was increased in the dose group of 16 mg/kg and above (Supplementary Table S3). Besides, doses ≥21.0 mg/kg demonstrated higher incidences of grade ≥2 rash: 43%–67% (21–28 mg/kg) vs. 8% (16 mg/kg; Supplementary Table S7). One case of grade 3 rash events was seen at the 28.0 mg/kg every 2 weeks dose group. Considering that these TRAEs may significantly affect patients’ quality of life during long-term treatment, it is recommended that the maximum dose should not exceed 16 mg/kg. Based on a collaborative assessment of safety and pharmacologic analysis, the RP2D for izalontamab was determined as 9 to 16 mg/kg weekly.

### Efficacy

**Figure 3.**
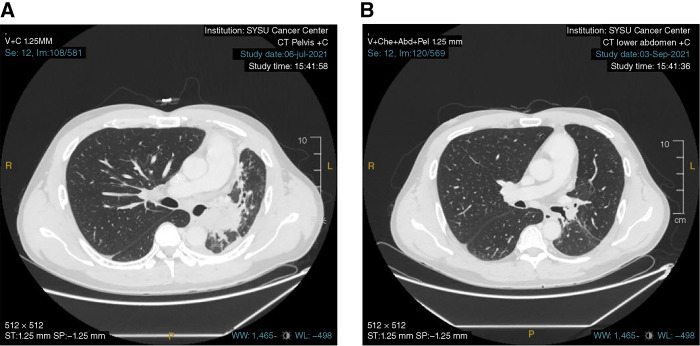
Typical radiographic images of the patient who achieved PR. **A** and **B,** A 41-year-old patient with HNSCC achieved PR after two cycles of izalontamab 16 mg/kg one dose per week treatment. SYSU Cancer Center, Sun Yat-sen University Cancer Center.

Of 57 evaluable patients, the confirmed ORR was 4% (2/57), the DCR (PRs and stable disease) was 37% (21/57), and the median PFS (95% CI) was 1.9 (1.7–2.8) months (Supplementary Figs. S2 and S3). Two PRs were observed. One patient with squamous cell lung carcinoma (no actionable genomic alterations) progressed on ACC006 (itraconazole) plus paclitaxel/carboplatin but achieved a PR after 2 months of izalontamab (6.0 mg/kg weekly) treatment, confirmed at 4 months. One patient with HNSCC, refractory to multiple prior therapies (including nab-paclitaxel/bleomycin/cisplatin and gemcitabine/S-1/sintilimab), achieved a PR after two courses of izalontamab (16 mg/kg weekly) and continued treatment for 9 months (Fig. 3). Eighteen patients had stable disease, including NSCLC (*n* = 17) and colorectal cancer (*n* = 1).

A total of 22 patients were able to obtain pretreatment specimens to explore the relationship between EGFR and HER3 expression and activity. The mean H-score for HER3 expression was 26.6, and the mean H-score for EGFR expression was 204.7. In patients with stable disease (*n* = 2) and progressive disease (*n* = 17), the mean H-score values of HER3 expression were 0 and 260; the mean H-score values of EGFR expression were 33.4 and 195.2, respectively. Responses were observed regardless of the EGFR or HER3 expression level (Supplementary Fig. S4).

## Discussion

In this phase I study, izalontamab, a novel EGFR × HER3 bispecific antibody, demonstrated a manageable safety profile and showed preliminary antitumor activity in patients with advanced epithelial tumors. Based on the comprehensive safety and PK assessment, the RP2D was preliminarily recommended as 9 to 16 mg/kg weekly administered. Further phase II and III studies are ongoing to explore the safety, optimal dosing for different tumors, and efficacy in monotherapy or combination strategies.

The main AEs of izalontamab were rash and paronychia, consistent with previous reports of expected on-target toxicities related to the EGFR inhibitors (12, 13). Skin rash is the most common target-related toxicity of EGFR antibodies, occurred in 42% of patients, lower than previously reported rates for EGFR/HER3 bispecific antibodies (14, 15) and cetuximab (80%–86%; refs. 16, 17). Grade ≥3 AEs were infrequent, supporting potential combination therapies. Infusion-related reactions, commonly observed in bispecific antibody therapies (20%–67%; refs. 18, 19), occurred in 23% of cases in this study, mostly grade 1 to 2 and manageable. Overall, izalontamab exhibited a favorable safety profile with low frequencies of AE-related dose modification and discontinuation.

In our study, izalontamab demonstrated nonlinear PK, which is consistent with its preclinical findings and previously reported EGFR/HER3 bispecific antibodies (11, 15). Izalontamab demonstrates a half-life of about 4 to 5 days, supporting a weekly dosing schedule. Izalontamab exhibits more rapid clearance at lower doses, whereas at doses ≥6 mg/kg, the clearance reached dose-independent steady-state kinetics, indicating target saturation. This is aligning with the PK profiles of reported EGFR-targeting mAbs and bispecific antibodies consistent with the saturation of target-mediated drug dispositions (15, 20). The C_max_ of 21 to 28.0 mg/kg is high, exceeded 2.5-fold that of 9 mg/kg, raising concerns about potential on-target toxicity. Notably, doses ≥21.0 mg/kg demonstrated significantly higher incidences of grade ≥2 rash. These TRAEs at higher doses may reduce patient quality of life and affect the tolerability of long-term treatment. Based on these PK and safety findings, the RP2D range is 9 to 16 mg/kg. Future studies exploring dose optimization in different tumor types use single agent or as combination regimen.

Historically, MEHD7945A, a dual-targeting EGFR/HER3 antibody, demonstrated promising efficacy in epithelial tumors (14, 21); however, its development was halted during early clinical stages because of toxicity concerns. In this study, izalontamab exhibited a favorable safety profile at doses of 9 to 16 mg/kg weekly, supporting its potential for long-term use and combination therapies. An objective response was observed in a patient with lung cancer. Phase II data demonstrated promising antitumor activity of izalontamab combined with docetaxel in patients with advanced NSCLC resistant to first-line anti–PD-1/L1 therapy, especially those without actionable oncogenic alterations (22). Phase III trials evaluating this combination are ongoing (NCT05943795).

The design of bispecific antibody–drug conjugates (ADC) offers the potential to target co-expressed antigens in tumors, thereby enhancing both selectivity and efficacy; however, the potential increased toxicity needs to draw more attention (23). Notably, the phase I study of BL-B01D1, a novel bispecific ADC incorporating izalontamab as the antibody component, demonstrated encouraging efficacy in patients with advanced solid tumors, achieving a response rate of 34% (60 of 174 patients; ref. 10). The most common toxicities included hematologic AEs (10), which are associated with the payload and manageable through proper supportive treatment; the incidence of EGFR/HER3-related target toxicity remains low, which aligns with the target toxicity profile observed for izalontamab. Currently, BL-B01D1 is being evaluated in several phase III clinical trials across different tumor types.

There were some limitations for this study. One limitation of our study is that this is a preliminary safety and efficacy evaluation of a phase I study, with a lack of randomization. Besides, due to the small number of patients and the multiple tumor types, the results of this study may not be fully representative, which will be confirmed in phase II and III trials. The biomarker analysis was performed in a relatively small cohort. Future studies are needed to explore and further investigate potential biomarkers associated with efficacy.

In conclusion, this phase I study demonstrates that izalontamab, a novel EGFR/HER3 bispecific antibody, can be safely administered with an acceptable toxicity profile in patients with advanced epithelial tumors. These findings also provide insights into further development of the ADC BL-B01D1, which shares the same antibody but adds a cytotoxic payload. A phase III study evaluating izalontamab in combination with docetaxel in NSCLC is currently underway.

## Supplementary Material

Supplementary Methods S1 Supplementary Text. Inclusion and exclusion criteria

Supplementary Figure S1 Supplementary Fig. S1 Pharmacokinetics of Izalontamab

Supplementary Figure S2 Supplementary Fig. S2 Swimmer plots of responses to treatment and the duration of treatment in efficacy analysis set.

Supplementary Figure S3 Supplementary Fig. S3 The waterfall plot depicts the best objective responses concerning the tumor size in the efficacy analysis set.

Supplementary Figure S4 Supplementary Fig. S4 The relationship between baseline EGFR/HER3 expression and best response in patients

Supplementary Table S1 Supplementary Table S1. Baseline clinical characteristics in different dose groups

Supplementary Table S2 Supplementary Table S2. Representativeness of Study Participants

Supplementary Table S3 Supplementary Table S3. Adverse events in different dose groups

Supplementary Table S4 Supplementary Table S4. Treatment-emergent adverse events of Izalontamab

Supplementary Table S5 Supplementary Table S5. Pharmacokinetic Parameter Summary of Izalontamab (Visit C1D22)

Supplementary Table S6 Supplementary Table S6. Summary of anti-drug antibody

Supplementary Table S7 Supplementary Table S7. Details of Rash and Paronychia at different doses

## Data Availability

The data supporting the findings of this study are included in the article and its Supplementary Information files. Clinical datasets have been deposited in the Research Data Deposit platform (https://www.researchdata.org.cn, RDDA2025546313); all datasets have been anonymized to ensure patient confidentiality and comply with relevant legal and ethical requirements. Data requests should be directed to the corresponding author and clearly reference the dataset ID RDDA2025546313. Requests will be reviewed by the corresponding authors, the Sun Yat-sen University Cancer Center Institutional Review Board, and Sichuan Baili Pharmaceutical Co., Ltd. to ensure compliance with intellectual property protection and confidentiality agreements. Due to proprietary restrictions, additional data are not publicly accessible, but requests can be directed to the corresponding author as outlined above.
