# Supplementary material for: Izalontamab (SI-B001), a Novel EGFRxHER3 Bispecific Antibody in Patients with Locally Advanced or Metastatic Epithelial Tumor: Results from First-in-Human Phase I/Ib Study
Source: Clin Cancer Res. 2025 Apr 21;31(21):4438–45. doi: 10.1158/1078-0432.CCR-25-0206 (PMC12580768; doi:10.1158/1078-0432.CCR-25-0206)
Supplement: Supplementary Methods S1 — Supplementary Text. Inclusion and exclusion criteria [file ccr-25-0206_supplementary_methods_s1_suppms1.pdf]

**Supplementary Text. Inclusion and exclusion criteria**

**Inclusion Criteria**

1. Signs the informed consent voluntarily and follow the program requirements;
2. Either Sex;
3. Age: ≥18 years and ≤75 years（phase Ia）; ≥18 years（phase Ib）;
4. Has a life expectancy of ≥ 3 months;
5. For patients in Phase Ia:

   Has a pathologically and/or cytological documented locally advanced or metastatic epithelial tumors that are cannot be cured or for which no standard therapy is available;

   For patients in Phase Ib:

   a. pathologically and/or cytological documented locally advanced or metastatic NSCLC patients who have progressed or are intolerant to PD-1/PD-L1 therapy, and are indicated by genetic sequencing to have EGFR and ALK wild-type, or EGFR exon 20 insertion mutation, or NRG1 fusion. If the subject is unable to provide a genetic sequencing report within six months, patients with histological adenocarcinoma subtypes should be excluded, and ctDNA testing should be completed before withdrawal from the study.

   b.Has a pathologically and/or cytological documented locally advanced or metastatic colorectal cancer who have failed or are intolerant to standard therapy , and are indicated by gene sequencing to have RAS wild-type if the subject cannot provide a genetic sequencing report within one year, subjects with right-sided tumor subtype must to be excluded, and ctDNA testing should be completed before withdrawal from the study;

   c.Has a pathologically and/or cytological documented locally advanced or metastatic squamous cell carcinoma of the head and neck （excluding nasopharyngeal carcinoma） who have progressed or are intolerant to immunotherapy;

   d.Has a pathologically and/or cytological documented locally advanced or metastatic esophageal squamous cell carcinoma，who currently have no

standard therapy available or are intolerant to standard therapy.

6. Agree to provide archived tumor samples or fresh samples from primary or metastatic sites within 6 months; If subjects are unable to provide tumor samples, they will be admitted after evaluation by the investigator if other admission criteria are met;
7. Has at least one measurable lesion based on RECIST V1.1;
8. Has an Eastern Cooperative Oncology Group performance status (ECOG PS) 0-1;
9. Toxicity of previous antitumor therapy has returned to Grade≤1 as defined by NCI- CTCAE V5.0 (except for alopecia);
10. Has not serious cardiac dysfunction, left ventricular ejection fraction≥50%;
11. Has adequate organ function before registration, defined as:

    a) Marrow Function: Absolute neutrophil count (ANC) ≥1.5×109/L, Platelet count ≥75×109/L, Hemoglobin (Hb)≥90 g/L;

    b) Hepatic function: Total bilirubin（TBIL≤1.5 ULN）, AST and ALT without liver metastasis ≤2.5 ULN, AST and ALT with liver metastasis ≤5.0 ULN;

    c) Renal function: Creatinine（Cr）≤1.5 ULN, or creatinine clearance(Ccr) ≥ 50 mL/min (According to the Cockcroft and Gault).

12. Coagulation function: international normalized ratio （INR） ≤1.5×ULN, and activated partial thromboplastin time （APTT）≤1.5ULN; (if the urine protein qualitative result is ≥ 2+, patients with 24-hour urine protein < 1g can be included in the study);
13. Urine Routine Test / Urine Protein Quantitation/24h: Urine protein result ≤1+(if Urinary protein≥2+,then urine protein/24h <1g is acceptable for enrollment);
14. For premenopausal women with childbearing potential, a pregnancy test must be taken within 7 days prior to the start of treatment. Serum or urine pregnancy must be negative and must be non-lactating. Adequate barrier contraceptive measures should be taken during the treatment and 6 months after the end of

treatment for all participants (regardless of male or female).

**Exclusion Criteria**

Patients screened for any of the following conditions were excluded from the study:

1. Chemotherapy, biological therapy, immunotherapy, radical radiotherapy, major surgery, targeted therapy (including small molecule inhibitor of tyrosine kinase), and other anti-tumor therapy within 4 weeks or 5 half-lives (whichever is shorter) prior to the first administration; mitomycin and nitrosoureas treatment within 6 weeks prior to the first administration; oral fluorouracil-like drugs such as S-1, capecitabine, or palliative radiotherapy within 2weeks prior to the first administration;
2. Within the 3 months prior to the first administration, Inactivated influenza vaccines are allowed to be received within 30 days prior to first administration, and live attenuated vaccines are not allowed.
3. Participants with history of severe heart disease within the six months prior to screening, such as: symptomatic congestive heart failure (CHF) Grade≥2 (CTCAE5.0), New York Heart Association (NYHA) Grade≥2 heart failure, history of transmural myocardial infarction, unstable angina pectoris etc;
4. Participants with prolonged QT interval (male QTc> 450 msec or female QTc> 470 msec), complete left bundle branch block, III grade atrioventricular block;
5. Active autoimmune diseases and inflammatory diseases, such as: systemic lupus erythematosus, psoriasis requiring systemic treatment, rheumatoid arthritis, inflammatory bowel disease and Hashimoto's thyroiditis, etc., except for type I diabetes, hypothyroidism that can be controlled only by alternative treatment, and skin diseases that do not require systemic treatment (such as vitiligo, psoriasis);
6. Other malignant tumors were diagnosed within 5 years prior to the first administration with the following exceptions: basal cell carcinoma of the skin, squamous cell carcinoma of the skin and/or carcinoma in situ after radical resection;
7. Patients with poorly controlled hypertension by two kinds of antihypertensive drugs (systolic blood pressure>150 mmHg or diastolic blood pressure>100 mmHg);

8. atients who have Grade≥3 lung disease defined according to NCI-CTCAE v5.0, or a history of interstitial lung disease (ILD);
9. Symptoms of active central nervous system metastasis. However, Patients with stable brain metastasis can be enrolled, with stability determined by the investigator;
10. Patients who have a history of allergies to recombinant humanized antibodies or human-mouse chimeric antibodies or any of the components of SI-B001;
11. Previous recipients of allogeneic hematopoietic stem cell transplantation or organ transplantation;
12. In the adjuvant (or neoadjuvant) treatment of anthracyclines, the cumulative dose of anthracyclines is> 360 mg/m2;
13. Human immunodeficiency virus antibody (HIVAb) positive, active tuberculosis, active hepatitis B virus infection (HBV-DNA copy number> 104) or active hepatitis C virus infection(HCV antibody positive and HCV-RNA > the lower limit of detection);
14. Patients with active infections requiring systemic treatment, such as severe pneumonia, bacteremia, sepsis, etc;
15. Other conditions that the investigator believes that it is not suitable for participating in this clinical trial;
16. Received another non-marketed investigational drug or treatment 4 weeks prior to fist administration of the investigational drug.
