# Supplementary material for: Izalontamab (SI-B001), a Novel EGFRxHER3 Bispecific Antibody in Patients with Locally Advanced or Metastatic Epithelial Tumor: Results from First-in-Human Phase I/Ib Study
Source: Clin Cancer Res. 2025 Apr 21;31(21):4438–45. doi: 10.1158/1078-0432.CCR-25-0206 (PMC12580768; doi:10.1158/1078-0432.CCR-25-0206)
Supplement: Supplementary Figure S1 — Supplementary Fig. S1 Pharmacokinetics of Izalontamab [file ccr-25-0206_supplementary_figure_s1_suppfs1.docx]

**Supplementary Fig. S1 Pharmacokinetics of Izalontamab**


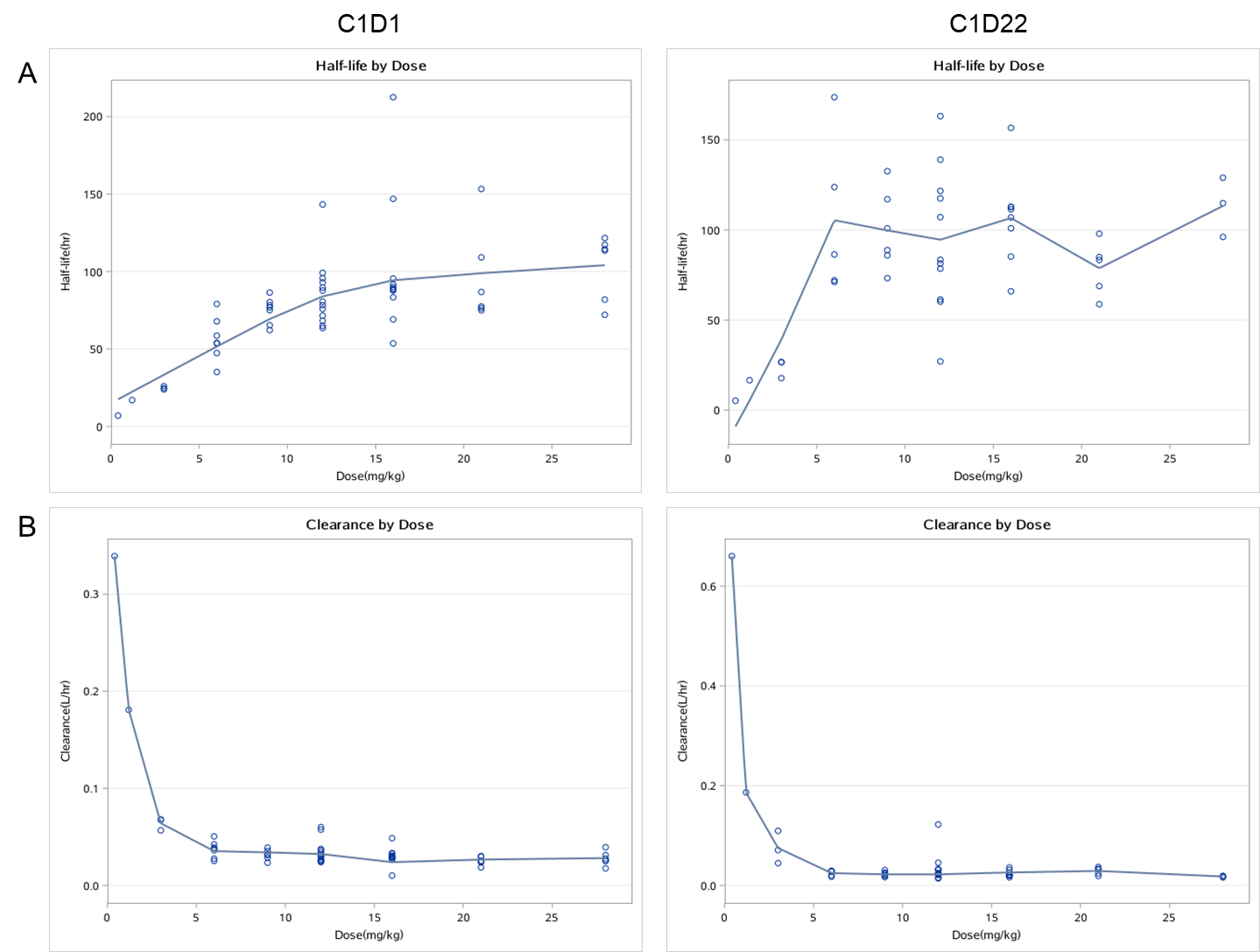


A, plot of half-life time. B, plot of serum clearance versus dose. C1D1, cycle 1 day 1; C1D22, cycle 1 day 22.
