# Supplementary material for: Izalontamab (SI-B001), a Novel EGFRxHER3 Bispecific Antibody in Patients with Locally Advanced or Metastatic Epithelial Tumor: Results from First-in-Human Phase I/Ib Study
Source: Clin Cancer Res. 2025 Apr 21;31(21):4438–45. doi: 10.1158/1078-0432.CCR-25-0206 (PMC12580768; doi:10.1158/1078-0432.CCR-25-0206)
Supplement: Supplementary Figure S3 — Supplementary Fig. S3 The waterfall plot depicts the best objective responses concerning the tumor size in the efficacy analysis set. [file ccr-25-0206_supplementary_figure_s3_suppfs3.docx]

**Supplementary Fig. S3 The waterfall plot depicts the best objective responses concerning the tumor size in the efficacy analysis set.**


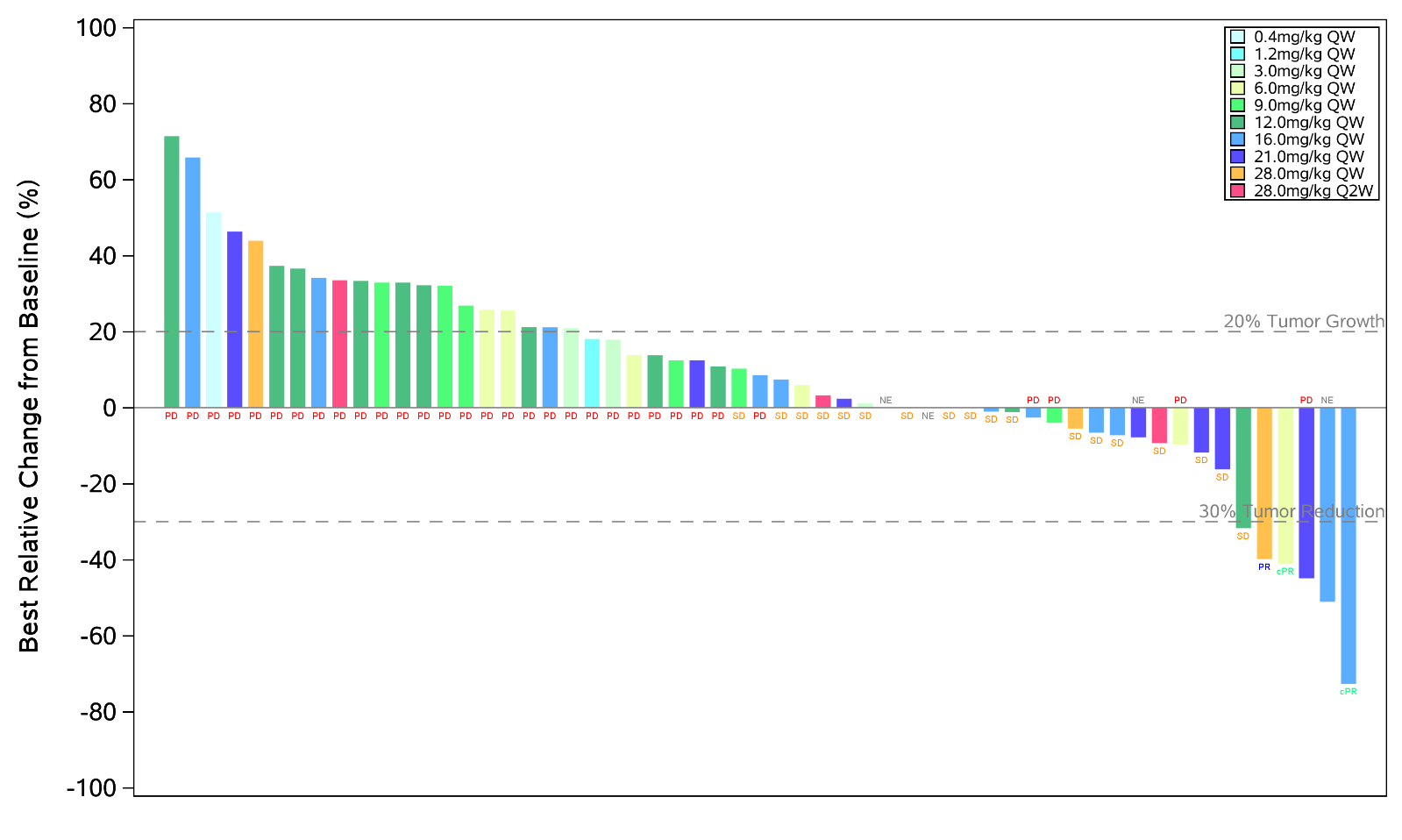


QW, weekly, Q2W, every two weeks, SD, stable disease, PD, progressive disease, NE, not evaluable.
