# Supplementary material for: Izalontamab (SI-B001), a Novel EGFRxHER3 Bispecific Antibody in Patients with Locally Advanced or Metastatic Epithelial Tumor: Results from First-in-Human Phase I/Ib Study
Source: Clin Cancer Res. 2025 Apr 21;31(21):4438–45. doi: 10.1158/1078-0432.CCR-25-0206 (PMC12580768; doi:10.1158/1078-0432.CCR-25-0206)
Supplement: Supplementary Figure S4 — Supplementary Fig. S4 The relationship between baseline EGFR/HER3 expression and best response in patients [file ccr-25-0206_supplementary_figure_s4_suppfs4.docx]

**Supplementary Fig. S4 The relationship between baseline EGFR/HER3 expression and best response in patients**


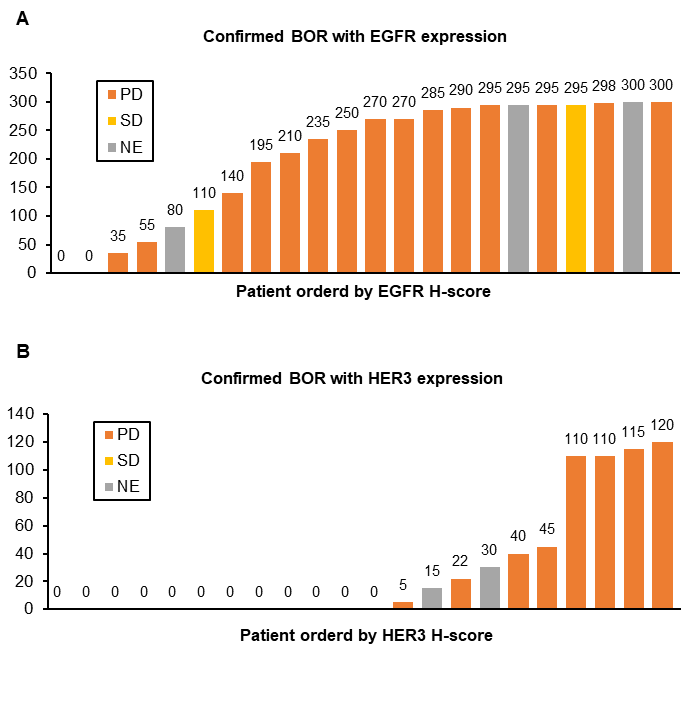


Pre-treatment specimens were avaliable from 22 patients to analyze the relationship between EGFR and HER3 expression and activity. The response to treatment is presented based on EGFR expression levels (A) and HER3 expressionlevels (B). BOR, best overall response, SD, stable disease, PD, progressive disease, NE, not evaluable.
