# Supplementary material for: Izalontamab (SI-B001), a Novel EGFRxHER3 Bispecific Antibody in Patients with Locally Advanced or Metastatic Epithelial Tumor: Results from First-in-Human Phase I/Ib Study
Source: Clin Cancer Res. 2025 Apr 21;31(21):4438–45. doi: 10.1158/1078-0432.CCR-25-0206 (PMC12580768; doi:10.1158/1078-0432.CCR-25-0206)
Supplement: Supplementary Table S1 — Baseline clinical characteristics in different dose groups [file ccr-25-0206_supplementary_table_s1_suppts1.docx]

**Supplementary Table S1. Baseline clinical characteristics in different dose groups**

|  | **QW  (N = 57)** | | | | | | | | | **Q2W  (N = 3)** |  |
| --- | --- | --- | --- | --- | --- | --- | --- | --- | --- | --- | --- |
|  | **0.4mg/kg  (N = 1)** | **1.2mg/kg  (N = 1)** | **3.0mg/kg  (N = 3)** | **6.0mg/kg  (N = 7)** | **9.0mg/kg  (N = 7)** | **12.0mg/kg  (N = 15)** | **16.0mg/kg  (N = 13)** | **21.0mg/kg  (N = 7)** | **28.0mg/kg  (N = 3)** | **28.0mg/kg  (N = 3)** | **Total (N = 60)** |
| **Age (years),** **Median (range)** | 48.0 (-) | 52.0 (-) | 55.0  (54.0-69.0) | 53.0  (35.0-61.0) | 59.0  (46.0-70.0) | 57.0  (32.0-74.0) | 56.0  (38.0-71.0) | 59.0  (23.0-69.0) | 59.0  (55.0-59.0) | 61.0  (60.0-66.0) | 57.0  (23.0 -74.0) |
| **Male** | 1 (100) | 1 (100) | 2 (67) | 3 (43) | 3 (43) | 11 (73) | 13 (100) | 7 (100) | 1 (33) | 3 (100) | 45 (75) |
| **Smoking History** |  |  |  |  |  |  |  |  |  |  |  |
| **Never** | 1 (100) | 0 | 3 (100) | 5 (71) | 4 (57) | 7 (47) | 4 (31) | 1 (14) | 2 (67) | 0 | 27 (45) |
| **Current** | 0 | 0 | 0 | 0 | 0 | 1 (7) | 1 (8) | 0 | 0 | 0 | 2 (3) |
| **Former** | 0 | 1 (100) | 0 | 2 (29) | 3 (43) | 7 (47) | 8 (62) | 6 (86) | 1 (33) | 3 (100) | 31 (52) |
| **ECOG** |  |  |  |  |  |  |  |  |  |  |  |
| **0** | 0 | 1 (100) | 2 (67) | 4 (57) | 1 (14) | 3 (20) | 2 (15) | 2 (29) | 0 | 2 (67) | 17 (28) |
| **1** | 1 (100) | 0 | 1 (33) | 3 (43) | 6 (86) | 12 (80) | 11 (85) | 5 (71) | 3 (100) | 1 (33) | 43 (72) |
| **Stage** |  |  |  |  |  |  |  |  |  |  |  |
| **III** | 0 | 0 | 0 | 0 | 0 | 1 (7) | 1 (8) | 1 (14) | 0 | 0 | 3 (5) |
| **IV** | 1 (100) | 1 (100) | 3 (100) | 7 (100) | 7 (100) | 14 (93) | 12 (92) | 6 (86) | 3 (100) | 3 (100) | 57 (95) |
| **Prior line of therapy, n(%)** |  |  |  |  |  |  |  |  |  |  |  |
| **1L** | 0 | 0 | 0 | 2 (29) | 0 | 3 (20) | 3 (23) | 0 | 0 | 1 (33) | 9 (15) |
| **2L** | 0 | 0 | 1 (33) | 1 (14) | 1 (14) | 6 (40) | 4 (31) | 1 (14) | 0 | 1 (33) | 15 (25) |
| **3L and above** | 1 (100) | 1 (100) | 2 (67) | 4 (57) | 6 (86) | 6 (40) | 6 (46) | 6 (86) | 3 (100) | 1 (33) | 36 (60) |

Note: Data are n (%). QW, weekly, Q2W, every two weeks.
