# Supplementary material for: Izalontamab (SI-B001), a Novel EGFRxHER3 Bispecific Antibody in Patients with Locally Advanced or Metastatic Epithelial Tumor: Results from First-in-Human Phase I/Ib Study
Source: Clin Cancer Res. 2025 Apr 21;31(21):4438–45. doi: 10.1158/1078-0432.CCR-25-0206 (PMC12580768; doi:10.1158/1078-0432.CCR-25-0206)
Supplement: Supplementary Table S2 — Representativeness of Study Participants [file ccr-25-0206_supplementary_table_s2_suppts2.docx]

**Supplementary Table S2. Representativeness of Study Participants**

| Cancer type(s)/subtype(s)/stage(s)/condition | Locally Advanced or Metastatic Lung Cancer and other Epithelial Tumors |
| --- | --- |
| Considerations related to: | |
| Sex | In locally advanced or metastatic epithelial tumors, the sex distribution typically shows a higher prevalence in males compared to females, often with a ratio of approximately 2:1. |
| Age | The median age at the time of locally advanced or metastatic epithelial tumors diagnoses is around 60. |
| Race/ethnicity | In non-small cell lung cancer, the typical race distribution includes 79.6% White, 3.0% Black, 10.4% Asian or Pacific Islander, and others. |
| Geography | In Global, approximately 2,480,000 new cases of lung cancer were estimated in 2022, with 1,800,000 deaths. In China, 820,000 new cases and 710,000 deaths of lung cancer were estimated in 2020. |
| Other considerations | The incidence of lung cancer in China is the highest in the world. This study mainly included East Asian populations, which underrepresentation in trials limits evaluation of the impact of racial/ethnic- or ancestry-based differences in efficacy and toxicity. |
| Overall representativeness of this study | The age distribution of our studies was similar to the mean age distribution of locally advanced or metastatic epithelial tumors in the literature, with a median age of 70 years.  In this study, the population was predominantly East Asian, with 75% males and 25% females. |
