# Supplementary material for: Izalontamab (SI-B001), a Novel EGFRxHER3 Bispecific Antibody in Patients with Locally Advanced or Metastatic Epithelial Tumor: Results from First-in-Human Phase I/Ib Study
Source: Clin Cancer Res. 2025 Apr 21;31(21):4438–45. doi: 10.1158/1078-0432.CCR-25-0206 (PMC12580768; doi:10.1158/1078-0432.CCR-25-0206)
Supplement: Supplementary Table S3 — Adverse events in different dose groups [file ccr-25-0206_supplementary_table_s3_suppts3.docx]

**Supplementary Table S3. Adverse events in different dose groups**

|  | QW  (N = 57) | | | | | | | | | Q2W  (N = 3) |  | |
| --- | --- | --- | --- | --- | --- | --- | --- | --- | --- | --- | --- | --- |
|  | 0.4mg/kg  (N = 1) | 1.2mg/kg  (N = 1) | 3.0mg/kg  (N = 3) | 6.0mg/kg  (N = 7) | 9.0mg/kg  (N = 7) | 12.0mg/kg  (N = 15) | 16.0mg/kg  (N = 13) | 21.0mg/kg  (N = 7) | 28.0mg/kg  (N = 3) | 28.0mg/kg  (N = 3) | **Total (N = 60)** |  |
| TEAEs, n (%) | 1 (100) | 1 (100) | 3 (100) | 7 (100) | 7 (100) | 15 (100) | 13 (100) | 7 (100) | 3 (100) | 3 (100) | 60 (100) |  |
| Associated with treatment discontinuation | 0 | 0 | 0 | 1 (14) | 0 | 1 (7) | 1 (8) | 1 (14) | 0 | 0 | 4 (7) |  |
| Associated with treatment delay | 0 | 0 | 0 | 0 | 0 | 2 (13) | 1 (8) | 2 (29) | 0 | 0 | 5 (8) |  |
| Associated with dose reduction | 0 | 0 | 0 | 0 | 0 | 0 | 0 | 0 | 0 | 1 (33) | 1 (2) |  |
| Associated with death | 1 (100) | 0 | 0 | 0 | 0 | 0 | 2 (15) | 0 | 0 | 0 | 3 (5) |  |
| Grade ≥3 TEAE | 1 (100) | 0 | 0 | 3 (43) | 1 (14) | 3 (20) | 5 (39) | 4 (57) | 1 (33) | 2 (67) | 20 (33) |  |
| Treatment-Related TEAE (TRAE) | 1 (100) | 1 (100) | 2 (67) | 6 (86) | 5 (71) | 15 (100) | 13 (100) | 6 (86) | 3 (100) | 3 (100) | 55 (92) |  |
| Associated with treatment discontinuation | 0 | 0 | 0 | 1 (14) | 0 | 0 | 1 (8) | 1 (14) | 0 | 0 | 3 (5.0) |  |
| Associated with treatment delay | 0 | 0 | 0 | 0 | 0 | 1 (7) | 0 | 2 (29) | 0 | 0 | 3 (5.0) |  |
| Associated with dose reduction | 0 | 0 | 0 | 0 | 0 | 0 | 0 | 0 | 0 | 1 (33) | 1 (2) |  |
| Associated with death | 0 | 0 | 0 | 0 | 0 | 0 | 0 | 0 | 0 | 0 | 0 |  |
| Grade ≥3 TRAE | 0 | 0 | 0 | 1 (14) | 0 | 3 (20) | 2 (15) | 1 (14) | 0 | 2 (67) | 9 (15) |  |

Note: Data are n (%). TEAE, treatment-emergent adverse events TRAE, treatment-related adverse events, QW, weekly, Q2W, every two weeks.
