# Supplementary material for: Izalontamab (SI-B001), a Novel EGFRxHER3 Bispecific Antibody in Patients with Locally Advanced or Metastatic Epithelial Tumor: Results from First-in-Human Phase I/Ib Study
Source: Clin Cancer Res. 2025 Apr 21;31(21):4438–45. doi: 10.1158/1078-0432.CCR-25-0206 (PMC12580768; doi:10.1158/1078-0432.CCR-25-0206)
Supplement: Supplementary Table S4 — Treatment-emergent adverse events of Izalontamab [file ccr-25-0206_supplementary_table_s4_suppts4.docx]

**Supplementary Table S4. Treatment-emergent adverse events of Izalontamab**

|  | **All (N=60)** | | | |
| --- | --- | --- | --- | --- |
|  | **Grade 1** | **Grade 2** | **Grade 3+** | *All Grade* |
| Rash | 21 (35) | 5 (8) | 1 (2) | 27 (45) |
| Proteinuria | 18 (30) | 3 (5) | 0 | 21 (35) |
| Paronychia | 10 (17) | 7 (12) | 0 | 17 (28) |
| Anaemia | 12 (20) | 2 (3) | 2 (3) | 16 (27) |
| Constipation | 16 (27) | 0 | 0 | 16 (27) |
| Hypoalbuminaemia | 14 (23) | 1 (2) | 0 | 15 (25) |
| Pyrexia | 13 (22) | 2 (3) | 0 | 15 (25) |
| IRR | 6 (10) | 6 (10) | 2 (3) | 14 (23) |
| Lymphocyte count decreased | 5 (8) | 4 (7) | 5 (8) | 14 (23) |
| Haemoptysis | 11 (18) | 1 (2) | 1 (2) | 13 (22) |
| Stomatitis | 13 (22) | 0 | 0 | 13 (22) |
| Hypokalaemia | 9 (15) | 2 (3) | 0 | 11 (18) |
| Pruritus | 9 (15) | 2 (3) | 0 | 11 (18) |
| Abdominal Pain | 10 (17) | 0 | 0 | 10 (17) |
| Nausea | 6 (10) | 4 (7) | 0 | 10 (17) |
| Urinary occult blood-positive | 10 (17) | 0 | 0 | 10 (17) |
| Decreased appetite | 7 (12) | 2 (3) | 0 | 9 (15) |
| Vomiting | 5 (8) | 4 (7) | 0 | 9 (15) |
| Asthenia | 7 (12) | 0 | 1 (2) | 8 (13) |
| Haematuria | 8 (13) | 0 | 0 | 8 (13) |
| Hypomagnesaemia | 4 (7) | 3 (5) | 1 (2) | 8 (13) |
| Alanine aminotransferase increased | 7 (12) | 0 | 0 | 7 (12) |
| Hypophosphataemia | 7 (12) | 0 | 0 | 7 (12) |
| Milia | 5 (8) | 2 (3) | 0 | 7 (12) |
| Neutrophil count increased | 7 (12) | 0 | 0 | 7 (12) |
| Productive cough | 7 (12) | 0 | 0 | 7 (12) |
| Protein urine present | 7 (12) | 0 | 0 | 7 (12) |
| Aspartate aminotransferase increased | 6 (10) | 0 | 0 | 6 (10) |
| Back pain | 4 (7) | 2 (3) | 0 | 6 (10) |
| Blood albumin decreased | 6 (10) | 0 | 0 | 6 (10) |
| Blood cholesterol increased | 6 (10) | 0 | 0 | 6 (10) |
| Chest pain | 6 (10) | 0 | 0 | 6 (10) |
| Diarrhoea | 6 (10) | 0 | 0 | 6 (10) |
| Fibrin D dimer increased | 6 (10) | 0 | 0 | 6 (10) |
| Headache | 5 (8) | 1 (2) | 0 | 6 (10) |

Note: Data are n (%). Treatment-emergent adverse events in ≥10% of patients are shown in the table.
