# Supplementary material for: Izalontamab (SI-B001), a Novel EGFRxHER3 Bispecific Antibody in Patients with Locally Advanced or Metastatic Epithelial Tumor: Results from First-in-Human Phase I/Ib Study
Source: Clin Cancer Res. 2025 Apr 21;31(21):4438–45. doi: 10.1158/1078-0432.CCR-25-0206 (PMC12580768; doi:10.1158/1078-0432.CCR-25-0206)
Supplement: Supplementary Table S5 — Pharmacokinetic Parameter Summary of Izalontamab (Visit C1D22) [file ccr-25-0206_supplementary_table_s5_suppts5.docx]

**Supplementary Table S5. Pharmacokinetic Parameter Summary of Izalontamab (Visit C1D22)**

| **Parameters**  **Cohorts** | **λz**  **(1/hr)** | **t1/2**  **(d)** | **Tmax**  **(hr)** | **Cmax**  **(mg/L)** | **Ctrough**  **(mg/L)** | **AUC0-t**  **(hr*mg/L)** | **AUC0-∞**  **(hr*mg/L)** | **Vss**  **(L)** | **CLss**  **(L/hr)** |
| --- | --- | --- | --- | --- | --- | --- | --- | --- | --- |
| **0.4 mg/kg**  **(n=1)** | 0.1339 | 0.22 | 2.0 | 4.7 | 0.0 | 28.3 | 34.1 | 4.93 | 0.6601 |
| **1.2 mg/kg**  **(n=1)** | 0.0419 | 0.68 | 2.0 | 25.2 | 0.0 | 309.3 | 348.9 | 4.45 | 0.1864 |
| **3.0 mg/kg**  **(n=3)** | 0.0304  (24.7) | 0.98  (21.6) | 3.9  (49.6) | 60.6  (48.8) | 0.5  (173.2) | 2371.8  (71.9) | 2497.0  (66.0) | 2.41  (25.1) | 0.0749  (43.5) |
| **6.0 mg/kg**  **(n=5)** | 0.0074  (34.3) | 4.39  (41.5) | 4.0  (49.4) | 159.7  (21.8) | 31.1  (25.8) | 13996.6  (30.7) | 22387.9  (56.1) | 3.48  (16.5) | 0.0246  (22.1) |
| **9.0 mg/kg**  **(n=6)** | 0.0072  (21.3) | 4.15  (21.9) | 4.5  (38.1) | 264.1  (28.3) | 61.5  (22.4) | 22941.8  (23.9) | 33625.1  (26.5) | 3.16  (13.0) | 0.0227  (22.2) |
| **12.0 mg/kg**  **(n=11)** | 0.0092  (64.6) | 3.94  (41.6) | 2.8  (55.4) | 322.4  (31.7) | 78.8  (64.1) | 31401.1  (43.4) | 40802.6  (53.9) | 3.91  (38.5) | 0.0339  (85.8) |
| **16.0 mg/kg**  **(n=8)** | 0.0069  (26.1) | 4.44  (24.4) | 2.4  (44.1) | 502.5  (20.0) | 130.1  (26.5) | 42433.7  (46.2) | 69083.5  (31.3) | 3.67  (35.2) | 0.0234  (27.3) |
| **21.0 mg/kg**  **(n=5)** | 0.0091  (20.4) | 3.28  (19.3) | 2.6  (16.5) | 679.5  (44.9) | 138.4  (54.5) | 52214.6  (48.5) | 66907.3  (51.0) | 3.34  (36.9) | 0.0290  (25.3) |
| **28.0 mg/kg QW**  **(n=3)** | 0.0062  (15.0) | 4.72  (14.6) | 3.4  (67.5) | 854.6  (15.9) | 258．3  (35.1) | 69493.8  (11.6) | 107625.0  (2.9) | 2.94  (22.7) | 0.0178  (8.9) |

Note：Data are presented as mean (Coefficient of Variation%). C1D22, Cycle 1 day 22.
