# Supplementary material for: Izalontamab (SI-B001), a Novel EGFRxHER3 Bispecific Antibody in Patients with Locally Advanced or Metastatic Epithelial Tumor: Results from First-in-Human Phase I/Ib Study
Source: Clin Cancer Res. 2025 Apr 21;31(21):4438–45. doi: 10.1158/1078-0432.CCR-25-0206 (PMC12580768; doi:10.1158/1078-0432.CCR-25-0206)
Supplement: Supplementary Table S6 — Summary of anti-drug antibody [file ccr-25-0206_supplementary_table_s6_suppts6.docx]

**Supplementary Table S6. Summary of anti-drug antibody**

|  | QW  (N = 57) | | | | | | | | |  | Q2W  (N = 3) |  | |
| --- | --- | --- | --- | --- | --- | --- | --- | --- | --- | --- | --- | --- | --- |
|  | 0.4mg/kg  (N = 1) | 1.2mg/kg  (N = 1) | 3.0mg/kg  (N = 3) | 6.0mg/kg  (N = 7) | 9.0mg/kg  (N = 7) | 12.0mg/kg  (N = 15) | 16.0mg/kg  (N = 13) | 21.0mg/kg  (N = 7) | 28.0mg/kg  (N = 3) |  | 28.0mg/kg  (N = 3) |  | Total (N = 60) |
| **ADA positive at baseline, n (%)** | 0 | 0 | 0 | 1 (14.3) | 1 (14.3) | 1 (6.7) | 0 | 0 | 1 (33.3) |  | 0 |  | 4 (6.7) |
| **at least 1 positive post-baseline result, n (%)** | 0 | 0 | 0 | 0 | 0 | 0 | 0 | 0 | 0 |  | 0 |  | 0 |
| **negative post-baseline result, n (%)** | 0 | 0 | 0 | 1 (14.3) | 1 (14.3) | 1 (6.7) | 0 | 0 | 1 (33.3) |  | 0 |  | 4 (6.7) |
| **ADA negative at baseline, n (%)** | 1 (100) | 1 (100) | 3 (100) | 6 (85.7) | 6 (85.7) | 14 (93.3) | 13 (100) | 7 (100) | 2 (66.7) |  | 3 (100) |  | 56 (93.3) |
| **at least 1 positive post-baseline result, n (%)** | 0 | 0 | 1 (33.3) | 1 (14.3) | 0 | 0 | 1 (7.7) | 0 | 0 |  | 0 |  | 3 (5.0) |
| **negative post-baseline result, n (%)** | 1 (100) | 1 (100) | 2 (66.7) | 5 (71.4) | 6 (85.7) | 14 (93.3) | 12 (92.3) | 7 (100) | 2 (66.7) |  | 3 (100) |  | 53 (88.3) |

Data are n (%). ADA, anti-drug antibody.
