# Supplementary material for: Izalontamab (SI-B001), a Novel EGFRxHER3 Bispecific Antibody in Patients with Locally Advanced or Metastatic Epithelial Tumor: Results from First-in-Human Phase I/Ib Study
Source: Clin Cancer Res. 2025 Apr 21;31(21):4438–45. doi: 10.1158/1078-0432.CCR-25-0206 (PMC12580768; doi:10.1158/1078-0432.CCR-25-0206)
Supplement: Supplementary Table S7 — Details of Rash and Paronychia at different doses [file ccr-25-0206_supplementary_table_s7_suppts7.docx]

**Supplementary Table 7 Details of Rash and Paronychia at different doses**

|  | **Grade** | **6.0mg/kg QW** | **9.0mg/kg QW** | **12.0mg/kg QW** | **16.0mg/kg QW** | **21.0mg/kg QW** | **28.0mg/kg QW** | **28.0mg/kg Q2W** |
| --- | --- | --- | --- | --- | --- | --- | --- | --- |
|  |  | **(N = 7)** | **(N = 7)** | **(N = 15)** | **(N = 13)** | **(N = 7)** | **(N = 3)** | **(N = 3)** |
| **Rash** | **All Grades** | 2 (29) | 1 (14) | 9 (60) | 5 (39) | 3 (43) | 2 (67) | 2 (67) |
|  | **Grade ≥2** | 0 | 0 | 0 | 1 (8) | 3 (43) | 0 | 2 (67) |
| **Paronychia** | **All Grades** | 0 | 0 | 5 (33) | 5 (39) | 2 (29) | 2 (67) | 1 (33) |
|  | **Grade ≥2** | 0 | 0 | 2 (13) | 2 (15) | 1 (14) | 1 (33) | 1 (33) |

Note: Data are n (%). QW, weekly, Q2W, every two weeks.
